# Global affordability of fluoride toothpaste

**DOI:** 10.1186/1744-8603-4-7

**Published:** 2008-06-13

**Authors:** Ann S Goldman, Robert Yee, Christopher J Holmgren, Habib Benzian

**Affiliations:** 1 School of Public Health and Health Services, The George Washington University, Washington, DC, USA; 2 WHO Collaborating Centre, Radboud University Medical Centre, Nijmegen, The Netherlands; 3 FDI World Dental Federation, Ferney-Voltaire, France

## Abstract

**Objective:**

Dental caries remains the most common disease worldwide and the use of fluoride toothpaste is a most effective preventive public health measure to prevent it. Changes in diets following globalization contribute to the development of dental caries in emerging economies. The aim of this paper is to compare the cost and relative affordability of fluoride toothpaste in high-, middle- and low-income countries. The hypothesis is that fluoride toothpaste is not equally affordable in high-, middle- and low-income countries.

**Methods:**

Data on consumer prices of fluoride toothpastes were obtained from a self-completion questionnaire from 48 countries. The cost of fluoride toothpaste in high-, middle- and low-income countries was compared and related to annual household expenditure as well as to days of work needed to purchase the average annual usage of toothpaste per head.

**Results:**

The general trend seems to be that the proportion of household expenditure required to purchase the annual dosage of toothpaste increases as the country's per capita household expenditure decreases. While in the UK for the poorest 30% of the population only 0.037 days of household expenditure is needed to purchase the annual average dosage (182.5 g) of the lowest cost toothpaste, 10.75 days are needed in Kenya. The proportion of annual household expenditure ranged from 0.02% in the UK to 4% in Zambia to buy the annual average amount of lowest cost toothpaste per head.

**Conclusion:**

Significant inequalities in the affordability of this essential preventive care product indicate the necessity for action to make it more affordable. Various measures to improve affordability based on experiences from essential pharmaceuticals are proposed.

## Introduction

Globalization has provoked changes in many facets of human life, particularly in diet. Trends in the development of dental caries in population have traditionally followed developmental patterns where, as economies grow and populations have access to a wider variety of food products as a result of more income and trade, the rate of tooth decay begins to increase. As countries become wealthier, there is a trend to greater preference for a more "western" diet, high in carbohydrates and refined sugars. Rapid globalization of many economies has accelerated this process [1]. These dietary changes have a substantial impact on diseases such as diabetes and dental caries [2,3]. The cariogenic potential of diet emerges in areas where fluoride supplementation is inadequate [4]. Dental caries is a global health problem [5] and has a significant negative impact on quality of life, economic productivity, adult and children's general health and development. Untreated dental caries in pre-school children is associated with poorer quality of life, discomfort, and difficulties in ingesting food that can result in failure to gain weight and impaired cognitive development [6]. Since low-income countries cannot afford dental restorative treatment [7] and in general the poor are most vulnerable to the impacts of illness, they should be afforded a greater degree of protection.

By WHO estimates one third of the world's population have inadequate access to needed medicines primarily because they cannot afford them [8]. Despite the inclusion of sodium fluoride in the World Health Organization's Essential Medicines Model List [9], the global availability and accessibility of fluoride for the prevention of dental caries remains a global problem. The optimal use of fluoride is an essential and basic public health strategy in the prevention and control of dental caries, the most common non-communicable disease on the planet. Although a whole range of fluoride vehicles are available for fluoride use (drinking water, salt, milk, varnish, etc.), the most widely used method for maintaining a constant low level of fluoride in the oral environment is fluoride toothpaste. As one of the key components of the WHO endorsed *Basic Package of Oral Care *[10], the promotion of affordable and effective fluoride toothpaste is important for improving equity in oral health.

The promotion of brushing twice a day with fluoride toothpaste is based on strong scientific evidence [11,12]. The widespread use of fluoride toothpaste has been recognised as the single most important reason for the decline of dental caries in developed countries during the 1970s and 1980s [13]. An example is the United Kingdom where the only organized preventive program has been that of water fluoridation but that only about 9% of the UK population benefit from optimally fluoridated water [14]. The introduction of fluoride toothpaste is the major most likely contributing factor to the decline in caries witnessed in the United Kingdom although other confounding factors inevitably play a role. More recently, the decline in dental caries amongst school children in Nepal, a low-income country, has been attributed to improved access to affordable fluoride toothpaste in Nepal [15]. For many low-income nations, fluoride toothpaste is probably the only realistic population strategy for the control and prevention of dental caries since cheaper alternatives such as water or salt fluoridation are not feasible due to poor infrastructure and limited financial and technological resources. The use of topical fluoride e.g. in the form of varnish or gels for dental caries prevention is similarly impractical since it relies on repeated applications of fluoride by trained personnel on an individual basis and therefore in terms of cost cannot be considered as part of a population based preventive strategy.

Based on global estimates, about 500 million people utilize fluoride toothpaste, 210 million have access to fluoridated water, 40 million have access to fluoridated salt, and 60 million benefit from fluoride mouth rinses, tablets and clinically applied fluoride [5]. Taking into account the global population for 2007 is estimated to be 6.6 billion it can be assumed that only about 12.5% of the world's population benefit from the caries preventive possibilities of fluoride toothpaste.

The use of an efficacious fluoride toothpaste is largely dependent upon its socio-cultural integration in personal oral hygiene habits, availability and the ability of individuals to purchase and use it on a regular basis. The price of fluoride toothpaste is believed to be too high in some developing countries [16] and this might impede equitable access. In a survey conducted at a hospital dental clinic in Lagos, Nigeria 32.5% of the respondents reported that the cost of toothpaste influenced their choice of brands and 54% also reported that the availability of dentifrices influenced their choice [17]. WHO endorses the development and use of affordable fluoride toothpaste and defines *affordable *toothpaste as "one that is available at a price that allows people on low income to purchase it [18]." To date there have not been any attempts to quantify affordability or to suggest a reasonable retail price which consumers might pay for fluoride toothpaste; nor has there been any research to evaluate the effects of affordability, purchasing, and utilisation. The aim of this paper is to compare the cost and relative affordability of fluoride toothpaste in high-, middle- and low-income countries. The hypothesis is that fluoride toothpaste is not equally affordable in high-, middle- and low-income countries.

## Methods

### Study design

A cross-sectional survey of fluoride toothpaste brands and the retail cost was conducted between December 2005 and March 2006. Data was collected on a self-completion questionnaire that was distributed to dental associations, non-governmental oral health organisations and individuals around the world in 136 countries. They were asked to provide in a tabulated format the brand name, the retail price and the quantity/package size for as many brands that could be identified on the local retail market. Several international brands were specified to facilitate comparison. Since the price of toothpaste often differs according to size sold, the price for a packaging size closest to 100 g/100 ml was asked. In addition, information was sought on the cheapest available fluoride toothpaste.

### Data entry and statistical analysis

The study assumed the therapeutic dose of fluoride toothpaste to be a pea-sized amount (0.25 g). The annual cost of fluoride toothpaste in US dollars per person was based on the therapeutic dose used twice daily for a year, which amounts to 182.5 g of toothpaste [19].

Toothpaste prices were obtained in national currency units. The data for all brands reported were entered into Microsoft Excel and converted to the annual cost expressed in US dollars using international exchange rate data (xe.com). In order to examine affordability, the prices were first adjusted to the year 2003 because the economic indicators used for comparisons were available most completely for that year. The 2006 to 2003 price adjustment was done using the inflation, GDP deflators for the years 2003, and 2004, obtained from the World Bank World Development Indicators 2006 [20]. For 2005, the 2004 GDP deflator was used because the 2005 deflator was not yet available.

The adjusted 2003 price and economic indicator data were converted to a SAS dataset for calculation of the median price for each country as well as other statistics using PROC MEANS and PROC UNIVARIATE. Two sets of comparisons were made, one using all products for which prices were collected, the second using the four selected international (or multinational brands) and the brand available at the cheapest price only. The ratios were calculated to facilitate the analysis of affordability within countries and in order to make cross-country comparisons. Household final consumption expenditures is the indicator used to evaluate affordability within the countries [20]. The per capita 2003 household final consumption expenditures were calculated for the total population and by income group, to allow for evaluation of the results by population segments, e.g. the poorest 30%, 50%, and 70% of the population. The analysis of affordability expressed the cost of the annually recommended dose of fluoride toothpaste as a proportion of the available household expenditures required to purchase enough toothpaste for one person for one year at the lowest available price. Affordability was also evaluated by estimating the number of days of work required to buy the recommended dose for one person for one year using the country's per capita annual income (basis 250 working days).

The measure chosen for affordability was a ratio of the number of days needed to pay for one annual therapeutic dosage of toothpaste at the lowest price for the poorest 30% of the population. According to Health Action International (HAI) a medication costing more than the equivalent of one day's wages is considered unaffordable [21].

The data from this affordability comparison was ranked into high and low prices using the median number of days of household final consumption income needed to pay for one dosage of toothpaste using one day as the cut-off point.

## Results

A total of 136 countries were contacted and 45 countries responded. Prices were obtained for 360 toothpaste products priced in 45 countries: 15 low-income, 17 middle-income, and 13 high-income countries. Economic data were available for 40 countries only, eliminating 3 low-income and 2 middle-income countries from the analysis (317 products). Where only the chosen international brands and the most inexpensive toothpastes available were analysed, data from 39 countries were used for a total of 137 products.

**Figure 1:**
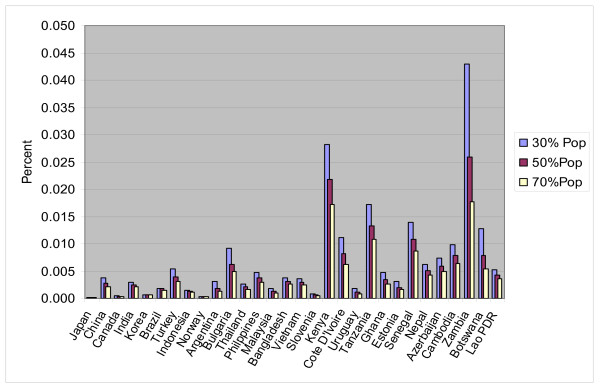
**Toothpaste (annual dosage) at lowest price as a proportion of annual household expenditures per capita**. Cost of one annual dosage of toothpaste at the lowest price as a proportion of annual household expenditures per capita by population group for selected countries.

Comparison of the ratio of the lowest and median toothpaste prices to household final consumption expenditures, by country, showed that as the per capita income decreases the proportion of annual per capita income required for the annual therapeutic dose of toothpaste increases (Figure 1). For the poorest 30% of the population, the ratio for all toothpaste products surveyed ranges from 0.015% (U.K) to 4.3% (Zambia) (median = 0.29; SD = 0.8, whereas the median for the total population is 0.07% (range 0.004%–0.8%; SD = 0.18%). A similar range and standard deviation were observed for prices for the selected international and the cheapest available brands only as proportion of annual household final consumption expenditures per capita for the poorest 30% of the population.

**Figure 2:**
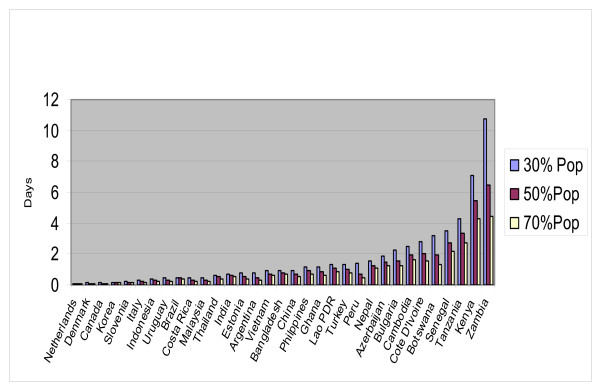
**Days of household expenditures to pay for toothpaste (one person, one year) at the lowest price**. Number of days of household expenditures required to pay for one annual dosage of toothpaste at the lowest price by country and population group. This figure includes countries for which the proportion was greater than 10% of a day of household expenditures.

Affordability is illustrated in Figure 2 with ratios of the lowest price of toothpaste as a proportion of one workday of per capita income for all brands for the four income distribution levels. Countries are ranked by household final consumption expenditures from highest on the left to lowest on the right. The resulting estimates for the number of workdays needed to pay for one annual dose of toothpaste per person at the lowest price for the poorest 30% of the population, range from 0.03 days in the United Kingdom to 9.34 days in Kenya (SD = 1.88), while for the same countries over the total population the range was 0.01–2. The range and standard deviation for the poorest 30% of the population is comparable for both the selected international brands and the lowest price brands.

When viewed by country category for all product brands surveyed in 40 countries, the prices for the poorest 30% of the population in each of 9 of the 12 (75%) of the lowest income countries were categorized as high, while prices in 6 of 15 (40%) of the middle-income countries were high. None of the high-income countries fell into the high category. As wealthier income groups were aggregated into the ratio the price category changed to the point where, for the total population toothpaste seemed expensive in only 4 (33%) of the low income countries and none of the middle income countries. Nonetheless for the lowest income countries the price remained in the high category in 7 of 12 countries for 70% of the population. In general, global brands seem to be more expensive than the generic brands.

### Limitations of the study

This investigation is not a comprehensive study on fluoride toothpaste affordability as only 24% of all World Bank member countries (184) participated in the survey. In addition, the data were predominantly collected from urban retail shops, chosen by convenience. Variations in retail cost of toothpaste and even of the same brands may occur within countries, between urban and rural markets and between countries due to natural factors (e.g. size of packaging, transportation costs) structural factors (e.g. local taxation and business regulations) and market conditions [22]. Larger retailers or wholesalers can charge lower prices, than small shops; whereas bargaining in street markets may result in lower prices.

The World Health Organization (WHO) and Health Action International (HAI) are field-testing a methodology for detailed country studies of affordability and costs of medicines within and between countries [21]. While this study was not designed using the WHO/HAI Medicine Prices protocol, in as much as possible the study adapted methodologies recommended in the protocol. The cross-sectional, multi-country nature of the study and prices obtained from retail outlets prevented the same comparisons.

### Global indicators used to facilitate comparisons

WHO/HAI suggests comparing "the cost of therapy with the daily wage of the lowest paid government worker [21]." The current study did not utilise wage information since it was not readily available; instead, household final consumption expenditures were used as a proxy for annual income.

The authors of this study recognise that all indicators, whether household data, income distribution or total health expenditure, possess limitations which make cross-country comparisons difficult. Therefore, future assessments of fluoride toothpaste affordability may benefit from the application of 'research triangulation'[23] as well as the use of multiple indices to further investigate this issue.

Ratios were calculated for the poorest 30%, 50% and 70% of the population and the total population. The ratios for the poorest 30% and the total population are reported to provide a sense of the difference in impact on the total population, compared to the strata of the poorest sector of the population with the greatest health needs and least access to services, including health and dental services. Access to a preventive measure like fluoride toothpaste has a potentially huge positive impact on these populations.

## Discussion

### Inequities in global affordability of fluoride toothpaste

The results of this study clearly demonstrate significant inequities in the affordability of fluoride toothpaste. There is a general trend where the poorer the country, the larger the proportion of the household expenditure that is needed to pay for one annual dosage of toothpaste for one person. In the 13 high-income nations the cost of toothpaste represents less than one percent of per capita household consumption expenditures, ranging from 0.004% to 0.041%. Toothpaste products surveyed in the middle- and low-income countries showed the proportion of household expenditure required to acquire one annual therapeutic dose of toothpaste is considerably larger and variable.

Two publications connected to the WHO-HAI studies state that a treatment regimen costing one or five days is considered expensive, and that these numbers are debatable [24]. While fluoride toothpaste is considered essential for the prevention of dental decay and its use should be part of daily hygiene, it should be more accessible and cheaper than life-saving medicines. On the basis of the survey results we suggest that a different metric is needed to establish a threshold for affordability, wherein the ability to pay of the poorest income groups, as well as a viable sales price are taken into account.

### Measures to reduce the costs of fluoride toothpaste

#### Equity Pricing

Equity pricing is based on the principle that the poor should pay less for, and have better access to an effective preventive product. The price of fluoride toothpaste should be fair, equitable and affordable, even for poor communities. The same brand of toothpaste should be available at different prices in different countries in accordance with the peoples' purchasing power.

#### Removal of taxation and tariffs

Taxes and tariffs on fluoride toothpaste sometimes significantly contribute to higher prices, lower demand and inequity since they target the poor. Toothpastes are usually classified as a cosmetic product and as such often highly taxed by governments. For example, various taxes such as excise tax, VAT, local taxes as well as taxation on the ingredients and packaging contribute to 25% of the retail cost of toothpaste in Nepal and India, and 50% of the retail price in Burkina Faso. In many developing countries essential preventive products, such as insecticide-treated mosquito nets, vaccines, contraceptives and oral rehydration salts, are exempt from import taxes or benefit from partial tax relief [25]. Olcay and Laing [24] found that pharmaceutical tariffs could be eliminated without adversely impacting on government revenue or industrial policy. There is also a significant negative relationship between the levels of tariffs and access to essential medicines. Analysis suggests that a 1% reduction in taxation will increase access to essential medicines by approximately 1% [26]. These findings may also be valid for fluoride toothpastes; hence, WHO continues to recommend the removal taxes and tariffs on fluoride toothpastes [5,27]. Any lost revenue can be restored by higher taxes on sugar and high sugar containing foods [28], which are common risk factors for dental caries, coronary heart disease, diabetes and obesity [29]. Along with tax relief on quality fluoride toothpaste, taxation of non-fluoride toothpaste, which has little preventive properties [30,31] would encourage consumers to make 'healthy choices the easy choices'. Any savings from tax relief on fluoride toothpastes must however be passed on to the customer.

#### Generic competition

Generic competition has been a powerful strategy for reducing drug prices and may have the same potential for increasing the availability and affordability of toothpastes. During the first half of the 1980s, world market prices for drugs on the WHO Model List fell by 40% through increased demand and competition [32]; while in Brazil the price of AIDS drugs fell by 82% over 5 years as a result of generic competition. In Myanmar, generic fluoride toothpaste is manufactured and distributed by the government – it is 3.5 times less expensive than the most expensive imported brand [33]. Social marketing has been successful in the prevention of HIV/AIDS and malaria [34,35] and has been proposed for increasing the availability and affordability of fluoride toothpaste [36].

#### Encouraging local production

The production of toothpaste within a country has the potential to make fluoride toothpaste more affordable than imported products. In Nepal, fluoride toothpaste was limited to expensive imported products. However, due to successful advocacy for locally manufactured fluoride toothpaste, the least expensive locally manufactured fluoride toothpaste is now 170 times less costly than the most expensive import [37]. In the Philippines, local manufacturers are able to satisfy consumer preferences and compete against multinationals by discounting the price of toothpaste by as much as 55% against global brands; and typically receive a 40% profit margin compared to 70% for multinational producers [38].

#### Inexpensive ingredients and packaging

Approximately 40% of the cost of production of toothpaste is related to the packaging, another 40% to the ingredients and 20% to labour [38]. High quality low cost fluoride toothpaste can be produced using (cheaper) precipitated calcium carbonate without interfering with the in vitro anti-caries efficacy [39]. Many countries use sachet packaging (10 ml) which make fluoride toothpaste more affordable to the poor who cannot afford a one-time expenditure for a larger quantity.

In order to achieve these measures, advocacy by international health organisations such as the WHO and the FDI World Dental Federation, as well as national advocacy by oral health stakeholders, is required in order to:

• Transform government policies and regional trade policies to eliminate taxation of quality fluoride toothpaste;

• Encourage generic and local production of affordable fluoride toothpaste.

• Encourage multinational toothpaste manufacturers to implement differential pricing for poorer countries and reduce the cost of toothpaste through inexpensive packaging and cheaper ingredients;

## Conclusion

World experts at a conference on "Oral Health through Fluoride for China and Southeast Asia" on September 18–19, 2007, in Beijing, China, have confirmed that: "fluoride toothpaste remains the most widespread and significant form of prevention of and protection against tooth decay used worldwide. It is also the most rigorously evaluated vehicle for fluoride use" [40]. In view of the current extremely inequitable use of fluoride throughout countries and regions, all efforts to make fluoride and fluoride toothpaste affordable and accessible must be intensified. As a first step to addressing the issue of affordability of fluoride toothpaste in the poorer countries in-depth country studies should be undertaken to analyze the price of toothpaste in the context of the country economies.

## Competing interests

The authors declare that they have no competing interests.

## Authors' contributions

CJH, RY, and HB conceived and designed the study, and supervised data collection, AG analyzed the data. All authors participated in additional research as well as drafting and editing the manuscript.
